# Success in the DREAM3 Signaling Response Challenge Using Simple Weighted-Average Imputation: Lessons for Community-Wide Experiments in Systems Biology

**DOI:** 10.1371/journal.pone.0008417

**Published:** 2010-01-26

**Authors:** Neil D. Clarke, Guillaume Bourque

**Affiliations:** Genome Institute of Singapore, Singapore, Singapore; Center for Genomic Regulation, Spain

## Abstract

Our group produced the best predictions overall in the DREAM3 signaling response challenge, being tops by a substantial margin in the cytokine sub-challenge and nearly tied for best in the phosphoprotein sub-challenge. We achieved this success using a simple interpolation strategy. For each combination of a stimulus and inhibitor for which predictions were required, we had noted there were six other datasets using the same stimulus (but different inhibitor treatments) and six other datasets using the same inhibitor (but different stimuli). Therefore, for each treatment combination for which values were to be predicted, we calculated rank correlations for the data that were in common between the treatment combination and each of the 12 related combinations. The data from the 12 related combinations were then used to calculate missing values, weighting the contributions from each experiment based on the rank correlation coefficients. The success of this simple method suggests that the missing data were largely over-determined by similarities in the treatments. We offer some thoughts on the current state and future development of DREAM that are based on our success in this challenge, our success in the earlier DREAM2 transcription factor target challenge, and our experience as the data provider for the gene expression challenge in DREAM3.

## Introduction

The DREAM3 signal transduction challenge and its assessment are described in more detail elsewhere in this issue. [1] Briefly, two hepatocyte cell lines, one normal and one cancer, were each treated with one of seven stimuli, or left untreated. Simultaneously the cells were treated with one of seven protein kinase inhibitors, or left uninhibited. Altogether, there were 64 combinations of stimulus (or non-treatment) and kinase inhibitor (or non-treatment). For each of these combinations, measurements were made at two time points. There was also a completely untreated (time = 0) sample. For each of the seven stimuli, data involving one of the seven inhibitors was withheld by the organizers and made a target for prediction. Data for a different inhibitor was withheld for each of the seven stimuli. For each combination of stimulus and inhibitor to be predicted, values were required for the normal and cancer cell lines, and for each of the two time points. For each combination of stimulus, inhibitor, cell line and time point, measurements were provided for 20 cytokines and 17 phosphoproteins. The cytokine and phosphoprotein predictions were assessed separately.

## Methods

**Figure 1:**
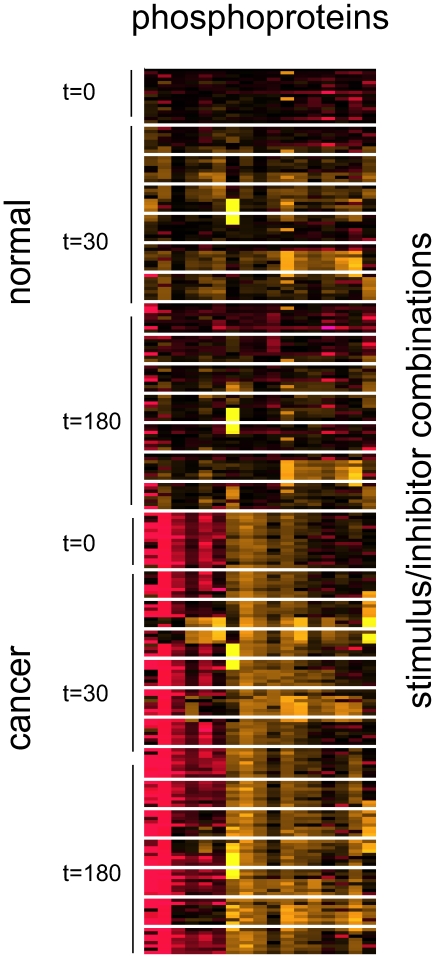
Visualization of the data provided to predictors for the phosphoprotein sub-challenge. Phosphoprotein levels were been normalized such that values above the median for all values are yellow and those below the median are red. Each column is one of the phosphoproteins, clustered based on similarity in expression. Rows correspond to experiments, sorted in an arbitrary hierarchical manner (cell type, time point, stimulus type, and inhibitor type). The white rows that appear to subdivide the dataset represent the missing data to be predicted.

To get a feel for what might work, we started by simply visualizing the data. An example of what we tried is shown in Figure 1 for the phosphoprotein set. Based on such visualizations, it was clear that different combinations of stimulus and inhibitor gave rise to similar profiles of phosphoproteins and cytokines. This implied that we might be able to directly impute the missing data points.

**Figure 2:**
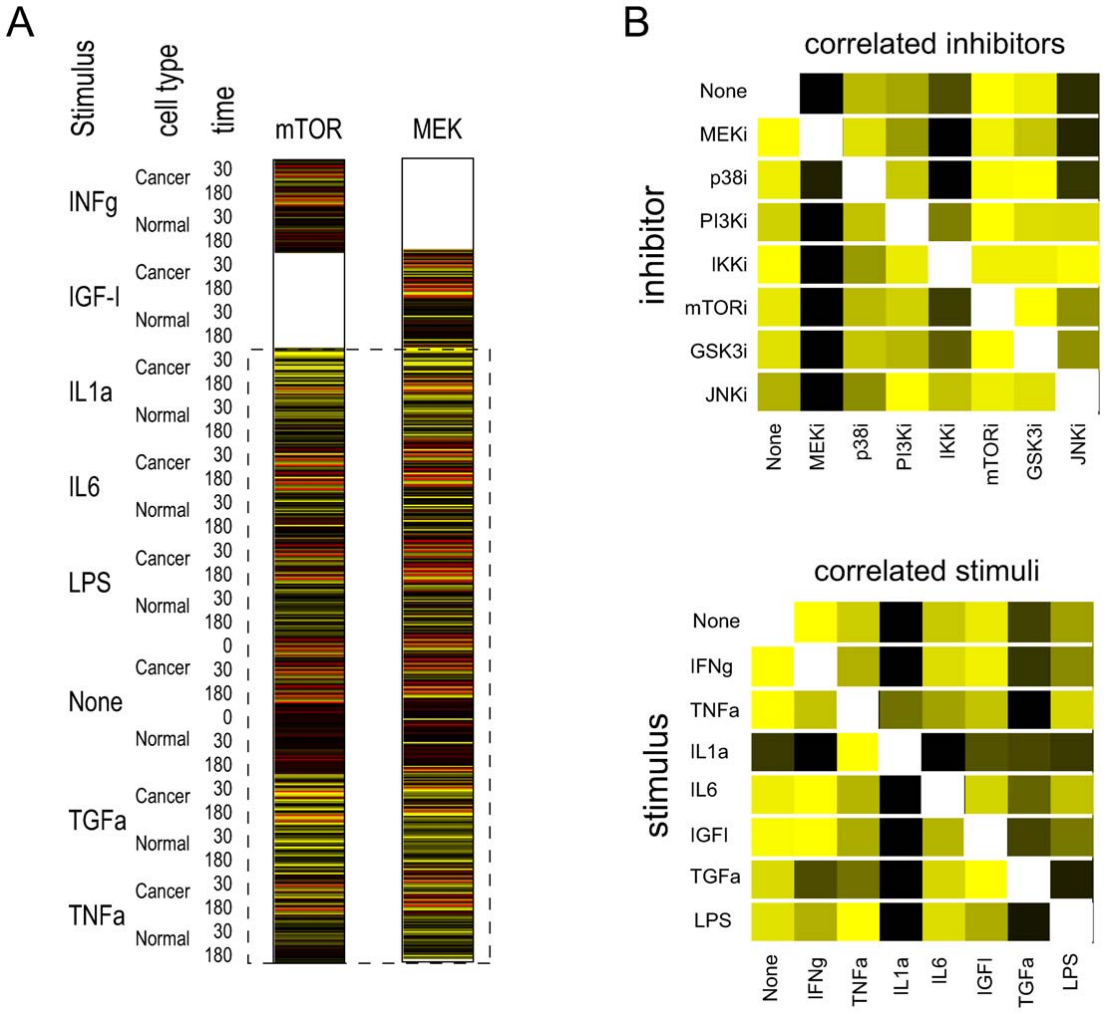
Determination of weights for calculating the weighted averages of similar experiments. (A) Example of how correlations between inhibitors and stimuli were calculated. The two colored columns represent the vector of phosphoprotein values obtained under all experimental conditions, sorted in an arbitrary but defined way. In the case of the mTOR inhibitor, data for the IGF-I stimulus is missing; these data are to be predicted. Similarly, in the case of the MEK inhibitor, data for the INFg stimulus is missing. The data in common (dashed box) was used to calculate the Spearman rank correlation coefficient. (B) Graphic representation of the normalized correlation coefficients relating inhibitors (top) and stimuli (bottom). The matrices are asymmetric because correlation coefficients were separately normalized for each inhibitor (stimulus), setting the maximum in a row to 1 (yellow) and the minimum to 0 (black). Other values were based on the correlation coefficient, scaling linearly between the minimum and maximum values in the row.

In order to predict the data for a particular stimulus-inhibitor combination, we chose to use data from the other experiments that involved the same stimulus or the same inhibitor. However, we wished to weight the contributions from these experiments based on how similar their marker protein profiles were to the combination of interest. To that end, we first assessed all pairwise similarities of inhibitors and, separately, all pairwise similarities of stimuli. Figure 2 illustrates how this was done. For all inhibitors, a data vector was constructed by concatenating the values for all phosphoproteins (or cytokines) measured in the presence of that inhibitor. The order is arbitrary, but of course has to be the same for all inhibitors. For each pair of inhibitors, there are two blocks of data, corresponding to two stimuli, for which values are missing. However, this leaves data from five stimuli and from unstimulated cells that can be used for calculating correlation coefficients. This amounts to a total of 442 values in each vector in the case of the phosphoprotein predictions and 520 in the case of the cytokines. Data vectors analogous to those constructed for the inhibitors were constructed for the stimuli as well, and all subsequent references to what was done with inhibitors also applies to stimuli.

For each pair of inhibitors, we calculated the Spearman rank correlation coefficient based on the data vectors described above. Values missing from one data vector or the other were ignored. For each inhibitor, we then calculated a normalized correlation coefficient to express the similarity of each of the other inhibitors to that inhibitor. The inhibitor with the highest correlation to the inhibitor of interest was given a weight of 1, the inhibitor with the lowest correlation was given a weight of 0, and the weights for all other inhibitors were scaled between these according to their correlation coefficients. (Figure 2B). For each missing value to be predicted, we then averaged the values from the six experiments that used the same inhibitor, weighting as described above, plus the values from the six experiments that used the same stimulus, also weighted as described.

## Results and Discussion

By the criteria used by the organizers of DREAM, our group did well, and we did so with an exceptionally simple algorithm. The algorithm was developed by inspecting the data, surmising that a weighted average of related experiments would work reasonably well, and then implementing a scheme to do it that seemed sensible. We did a very limited validation using simulated missing data, which suggested that the scaling of weights was a sensible thing to do. However, we made no attempt to systematically optimize the method. The success of such a simple algorithm suggests that the data set as a whole is substantially redundant, and that the missing data are mostly well-determined by data from related experiments.

The importance of having challenges that assess our ability to predict real data is something that we advocated at the DREAM2 and DREAM3 meetings, and in a previous paper that describes our success with the DREAM2 transcription factor target challenge. [2] Interested readers will find in that paper a discussion about the potential for community self-deception when the distinction between data and the interpretation of data becomes blurred. “We believe we did well in the DREAM2 transcription factor target challenge, at least in small part, because we adopted a model for interpreting expression data that we knew that the organizers themselves subscribed to. We think this should be avoided wherever possible and applaud the decision to move towards challenges, such as this DREAM3 signal transduction challenge, in which data are predicted.

Having argued so strongly for objective, data-dependent assessments in the DREAM process, we were pleased to participate in DREAM3 not only as predictors in the signaling response challenge, but also as the providers of data for the gene expression challenge. In our challenge, much like the signaling response challenge, a large amount of data was provided under a set of related conditions (yeast genotypes), and predictors were asked to infer something about the data for a small subset of genes. One of the two groups that did best did make an attempt to include additional datatypes (i.e., ChIP-chip data) but our sense is that most if not all of the success in that challenge amounted to the imputation of missing data, albeit by methods more elaborate than what we did in the signaling response challenge.

Does the success of imputation, and the corresponding lack of new insights into biology, mean that data-driven assessments in DREAM are a failure? We don't think so. First, it is inevitable that imputation of some sort will be the superior method given a sufficient amount of relevant data. One solution might be to simply provide much less data, or to require predictors to extrapolate and not just interpolate. Second, it is not clear how well imputation is really doing, only that it performs significantly better than random. What are the predictions that we and others got wrong, and what could we have done to do better? Perhaps part of the assessment should be focused more on the problems with the predictions, rather than the low-hanging fruit that we are currently calling a success? Finally, we need to ask why we are making the predictions and whether the level of success is sufficiently good for that purpose. We are reminded here of the community-wide protein structure prediction experiments, CASP, and the category of predictions that is traditionally called homology modeling, but is now more commonly called template-based modeling. [3], [4] Even more than the imputation of data in the DREAM challenges, it can be a trivial result to conclude that a protein adopts a particular fold if there is sufficient information (in this case, sufficient sequence similarity to a protein of known structure). However, there would be great practical value in going further, and predicting the coordinates of the structure to an accuracy comparable to that which can be achieved experimentally. After nearly 15 years of objective assessment in CASP, the challenge remains unsolved. However, the problem has become better defined, the methods more robust, and the relevant data more abundant. We are optimistic that the same will happen with DREAM.
